# 7-Ketocholesterol promotes T cell migration through Ca^2+^-NFATc1 pathway-mediated F-actin polymerization and proinflammatory cytokine production in oral lichen planus

**DOI:** 10.3389/fimmu.2026.1682589

**Published:** 2026-02-06

**Authors:** Qin Jiang, Yu-Xi Tang, Gang Zhou

**Affiliations:** 1 State Key Laboratory of Oral & Maxillofacial Reconstruction and Regeneration, Key Laboratory of Oral Biomedicine Ministry of Education, Hubei Key Laboratory of Stomatology, School & Hospital of Stomatology, Wuhan University, Wuhan, China; 2 Department of Oral Medicine, School and Hospital of Stomatology, Wuhan University, Wuhan, China

**Keywords:** 7-Ketocholesterol, Ca^2+^-NFATc1 signaling pathway, F-actin polymerization, oral lichen planus, oxysterol, proinflammatory cytokines

## Abstract

**Background:**

Oral lichen planus (OLP) is a chronic T-cell-mediated inflammatory disorder of unknown etiology. Accumulating evidence has demonstrated elevated cholesterol levels in OLP, and its oxidation products --oxysterols have been implicated in T cell dysfunction. However, whether the oxysterol is involved in OLP pathogenesis remains to be fully elucidated.

**Methods:**

Metabolomics was performed to profile oxysterols in the plasma of OLP patients, followed by functional enrichment analysis. Single-cell RNA sequencing was utilized to characterize gene expression dysregulation in tissue-resident T cells isolated from OLP lesions. Flow cytometry, immunofluorescence, and qRT-PCR were collectively used to quantify Ca^2+^ concentration, cell apoptosis, protein expression, intracellular signaling, and gene transcription levels. Functional validation was conducted through a co-culture model and Transwell migration assays to assess the cytotoxic and migratory capacity of OLP T cells.

**Results:**

The oxysterol profiles were aberrant in OLP plasma, with marked accumulation of 7-ketocholesterol (7KC). Functional analysis identified significant enrichment of differential metabolites in androstenedione metabolism. 7KC upregulated the expression of cholesterol regulators (SREBP2/LXR) in OLP T cells. Pro-7-ketocholesterogenic gene sets were dysregulated in OLP tissues, with localized T cells exhibiting enriched Ca^2+^ -NFATc1 signaling and coordinated F-actin polymerization/*ITGAL* (LFA-1α) upregulation, positively correlating with migration signatures. Peripheral OLP T cells showed elevated Ca^2+^, nuclear NFATc1, F-actin polymerization, and LFA-1α, all of which, along with *ITGAL/IL1B/CCL4/IL6* levels, were further potentiated by 7KC treatment. 7KC was confirmed to enhance migrations of primary OLP T cells and OLP plasma-pretreated Jurkat T cells toward LPS-treated keratinocytes, without affecting keratinocyte apoptosis. Furthermore, CM4620-mediated blockade of Ca^2+^ -NFATc1 pathway in OLP T cells inhibited 7KC-induced NFATc1 activation, reduced the expressions of F-actin and its modulators *ACTB/DIAPH1*, and *IL1B/CCL4/IL6* gene expressions, with migration suppressions of both primary OLP T cells and OLP plasma-pretreated Jurkat T cells.

**Conclusions:**

7KC could promote T cell migration through Ca^2+^ -NFATc1 pathway-mediated F-actin polymerization and expression of *IL1B/CCL4/IL6* in OLP.

## 1 Introduction

Oral lichen planus (OLP) is a chronic immune-mediated oral mucosal disease with unknown etiology, frequently affecting middle-aged females, and has been recognized by the WHO as an oral potentially malignant disorder (OPMD) (1, 2). Based on the clinical presentation, OLP is classified into erosive (EOLP) and non-erosive (NEOLP) subtypes (3). The histopathological hallmarks of OLP include band-like lymphocytic infiltration in the lamina propria and liquefactive degeneration of basal keratinocytes (4). While the pathogenesis remains elusive, dysregulated T cell immunity is widely implicated in OLP progression, where cytokine- and adhesion molecule-mediated recruitment of T cells from peripheral blood to lesion sites drives keratinocyte apoptosis (5).

Emerging evidence has indicated dyslipidemia in OLP, with significantly elevated average levels of total cholesterol and low-density lipoprotein (LDL) cholesterol compared to healthy controls (6). Oxysterols are downstream metabolites of cholesterol oxidation, which can be generated through hydroxylase-mediated enzymatic and reactive oxygen species (ROS)-driven non-enzymatic pathways, while local oxidative stress, ROS and myeloperoxidase upregulated in OLP potentially created conditions for the non-enzymatic pathway (7–9). Dysregulated oxysterols in autoimmune diseases have been demonstrated to contribute to the pathogenesis by regulating the migration, inflammatory responses, intercellular communication, and lipid homeostasis of T cells (7, 10). 7-Ketocholesterol (7KC), a major oxidized LDL component synthesized predominantly through non-enzymatic pathway, was elevated in various inflammatory diseases such as psoriasis, inflammatory bowel diseases (IBD) and multiple sclerosis (MS) (10, 11). 7KC was positively correlated with the disease severity of MS, and exacerbated the infiltration of inflammatory cells and the secretion of cytokines such as TNF-α in psoriasis (10). However, aberrant oxysterols and their roles in OLP remain uncharacterized.

Oxysterols such as 7KC have been reported to trigger intracellular Ca^2+^ flux in T cells, activating calcineurin-dependent dephosphorylation of the nuclear factor of activated T cells (NFAT) and its subsequent nuclear translocation, with the nuclear factor of activated T cells, cytoplasmic 1 (NFATc1) isoform being critical for T cell activation and sustained effector functions (12, 13). Among Ca^2+^ channels, the CRAC channel plays a unique role in lymphocytes, regulating cell motility and the production of cytokines (14). Ca^2+^-NFAT pathway activity and Ca^2+^ concentration were elevated in T cells in multiple autoimmune disorders, positively correlated with disease susceptibility and activity (15). Inhibition of calcineurin suppressed T cell activation and inflammatory cytokine profiles (15). In addition, elevated intracellular Ca^2+^ and NFAT nucleation enhanced the affinity maturation of LFA-1 and the F-actin polymerization, ultimately facilitating cell adhesion and migration (16–20). The oxysterol 7KC induced expression of proinflammatory cytokines, such as MIP-1β, TNF-α, IL-8, IL-1β, and IL-6, and adhesion molecules LFA-1α, while concurrently coordinating F-actin reorganization (21–26). Furthermore, calcineurin inhibitors have shown good efficacy in glucocorticoid-tolerant OLP, significantly reducing serum levels of IL-6 and IL-8 (27). The LFA-1α was positively expressed in almost all infiltrating lymphocytes in OLP lesions, and the culture supernatant of OLP-derived keratinocytes significantly induced the recombination of actin and the formation of pseudopodia in PBMC to promote migration (28, 29). The dysregulated cytokine milieu in OLP, characterized by elevated MIP-1β, IL-6, IL-1β, TNF-α and IL-8, played a pivotal role in the pathogenesis of OLP, and the upregulations of IL-1β and MIP-1β in OLP were demonstrated to promote T cell migration in our previous studies (5, 30–32). Therefore, targeting the Ca^2+^-NFATc1 axis in T cells, along with its downstream effectors F-actin reorganization, LFA-1α expression and proinflammatory cytokine networks, represents a rationally designed strategy to elucidate oxysterol-mediated pathogenesis in OLP.

In this study, we first quantified cholesterol metabolites in OLP, identifying 7KC as a significantly upregulated oxysterol, conducted functional annotation of differential metabolites, and validated the feedback effect of 7KC on the cholesterol metabolic SREBP2-LXR axis. Single-cell RNA sequencing data from NEOLP and EOLP tissues were later analyzed to characterize the potential conditions for 7KC production in local cellular subsets, evaluate the relative expression levels of the Ca^2+^-NFATC-F-actin/LFA-1α axis in local T cells and its correlation with migration gene set, and explore the function enrichment of local OLP T cells with high expression of Ca^2+^ signaling pathway. Subsequently, the abnormality of Ca^2+^-NFATc1-F-actin/LFA-1α axis in peripheral OLP T cells was studied, and the effect of 7KC on the Ca^2+^-NFATc1-F-actin/LFA-1α signaling axis and pro-inflammatory cytokines in primary OLP T cells and OLP plasma-pretreated Jurkat T cells was verified *in vitro* cell experiments. Transwell co-culture systems with primary OLP T cells or OLP plasma-preconditioned Jurkat T cells and LPS-pretreated keratinocytes were then constructed to study 7KC-mediated regulation of T cell migration and T cell-driven keratinocyte apoptosis. The blocker of Ca^2+^ channel CRAC was further employed to determine whether the effects of 7KC depend on Ca^2+^-NFATc1 signaling in OLP T cells.

## 2 Materials and methods

### 2.1 Participants and samples

All procedures in this research were conducted in compliance with the ethical principles outlined in the Declaration of Helsinki and approved by the Medical Ethics Committee of the School and Hospital of Stomatology at Wuhan University (Approval No. 2024A66). Peripheral blood samples from 40 individuals were obtained, comprising 19 patients diagnosed with OLP and 21 healthy volunteers. Detailed demographic and clinical profiles of the study cohort are provided in **Supplementary Table 1**. Written informed consent was secured from each participant prior to enrollment. OLP diagnoses were confirmed through both clinical examination and histopathological evaluation, adhering to WHO diagnostic standards, and all cases fulfilled the inclusion/exclusion criteria predefined by us (**Supplementary Table 2**). Disease severity was evaluated independently by two blinded examiners using the Reticular, Atrophic, and Erosive (RAE) scoring systems we proposed earlier (**Supplementary Table 3**).

### 2.2 PBMCs isolation, T cells purification and plasma separation

Heparin-coated vacuum collection tubes were used to prevent coagulation of the obtained blood sample. Peripheral blood mononuclear cells (PBMCs) were subsequently isolated via density-gradient centrifugation using Ficoll-Hypaque solution (TBD Science, China). After centrifugation, the PBMC layer was carefully extracted, rinsed twice with phosphate-buffered saline (PBS), and resuspended for downstream processing. Concurrently, plasma fractions from the supernatant were aliquoted and cryopreserved at −80 °C for further analyses and applications. T lymphocytes were then purified from PBMCs through negative selection employing the BD IMag™ Human T Lymphocyte Enrichment Set-DM coupled with the IMag™ magnetic separation platform. As shown in our previously published data, the T cell purity after negative sorting exceeded 98% (33).

### 2.3 Liquid chromatography-trandem mass spectrometry analysis of steroids

After thawing, plasma samples from the control (male-to-female ratio 1:1) and OLP (male-to-female ratio 1:3) cohort were vortexed (10 sec). Then, 100 μL aliquots were mixed with 400 μL methanol, vortexed (10 min), incubated on ice (10 min), and centrifuged (12,000 rpm, 5 min, 4°C). The supernatant (400 μL) was dried under nitrogen and reconstituted in 100 μL methanol, vortexed (5 min), centrifuged (12,000 rpm, 3 min, 4°C), and 80 μL supernatant was collected for LC-MS/MS analysis. The sample extracts were analyzed using an LC-ESI-MS/MS system (UPLC, ExionLC AD https://sciex.com.cn/; MS, QTRAP^®^ 6500+ System, https://sciex.com/). The analytical conditions were as follows, HPLC: column, Phenomenex Kinetex C18 1.7 µm, 100 mm × 2.1 mm i.d.; solvent system, 30% acetonitrile/water with 0.04% Acetic acid (A), 50% acetonitrile/isopropanol with 0.04% Acetic acid (B); The gradient was started at 5% B (0 min-1.0 min), increased to 90% B (1.0–10 min), maintained at 90% B (10-12.5 min), finally ramped back to 5% B (12.6–15 min); flow rate, 0.35 mL/min; temperature, 40°C; injection volume: 5 μL. HPLC-grade solvents (ACN, IPA, MeOH; Merck) and acetic acid (Sigma-Aldrich) were used. Standards (Olchemlm Ltd.) were prepared as 1 mg/mL MeOH stock solutions (stored at -20°C) and diluted to working concentrations prior to analysis. Milli-Q water was used throughout.

For ESI-MS/MS conditions, the AB6500+QTRAP^®^LC-MS/MS System with ESI Turbo Ion-Spray interface operated in positive mode (Analyst 1.6 software). The ESI source operation parameters were as follows: ion source, turbo spray; source temperature 550 °C; ion spray voltage (IS) 5500 V Positive; curtain gas (CUR) 35.0 psi; MRM transitions optimized for DP/CE and neurotransmitter-specific MRM monitoring.

Differential metabolites were screened using OPLS-DA VIP scores (biological replicates ≥ 3) and univariate analysis (|log2FC| ≥ 1, FC ≥ 2 or ≤ 0.5). The HCA(hierarchical cluster analysis) results and Pearson correlation coefficients (PCC) of differential metabolites were calculated using the core function in R and presented as heatmap. Identified metabolites were annotated using Kyoto Encyclopedia of Genes and Genomes (KEGG) compound database (http://www.kegg.jp/kegg/compound/), and then mapped to KEGG Pathway database (http://www.kegg.jp/kegg/pathway.html). Pathways with significantly regulated metabolites mapped to were then fed into MSEA (metabolite sets enrichment analysis), with significance determined by hypergeometric test’s P-Values. Furthermore, the differential metabolites were enriched based on the Human Metabolome Database (HMDB) and metabolite-related diseases were explored.

### 2.4 Flow cytometry

For the detection of transcription factors in 7KC-treated/untreated purified primary CD3^+^ T cells which were both pre-stimulated with CD3/CD28 antibodies, cell fixation, permeabilization, and washing procedures were performed using a transcription factor buffer kit (BD pharmingen, USA) following the standard protocol. Subsequently, cells were incubated with Alexa Fluor 594-conjugated SREBP2 and Alexa Fluor 488-conjugated LXRα/β antibodies (1:20, Biolegend, USA) at 4°C protected from light for 30 min.

For analysis of surface molecule LFA-1α, 7KC/CM4620-treated or untreated purified OLP/control T cells and plasma-pretreated Jurkat T cells, which were both pre-stimulated with CD3/CD28 antibodies, and purified primary T cells were incubated with FITC-conjugated anti-LFA-1α antibody (1:20, Thermofisher, USA) at 4°C for 30 min in the dark.

Following staining, cell suspensions (0.3 mL PBS) were subjected to flow cytometric analysis using a CytoFLEX LX (Beckman Coulter, Brea, USA). Instrument settings were optimized by adjusting voltage parameters and establishing appropriate compensation matrices. Cells were sequentially gated on FSC/SSC to exclude debris, on FSC-H/FSC-A to select singlets, and subsequently on a viability dye to discriminate live cells. Data acquisition and subsequent analysis were performed using the CytoFLEX LX (Beckman Coulter, Fullerton, CA, USA) for comprehensive data interpretation.

### 2.5 Single-cell raw data collection, processing and analysis

ScRNA-seq datasets (GSE211630) comprising 10× scRNA-seq data from 3 NEOLP samples, 2 EOLP samples and 1 normal oral mucosa sample were acquired from GEO database. Initial processing involved conversion to Seurat objects by the R software “Seurat” package, followed by quality filtering, where cells with low gene counts or elevated mitochondrial content were excluded. After data integration using the Harmony package, dimensionality reduction with Uniform Manifold Approximation and Projection (UMAP) and subsequent cell clustering using the FindClusters function were performed. Cell cluster annotation was performed using the SingleR package in R, with manual refinement based on canonical markers from published literature. Cell type-specific expression patterns of pro-7-ketocholesterogenic genes were spatially resolved through UMAP coordinate projection. Gene set enrichment scores were calculated using the “AddModuleScore” function implemented in the Seurat package. The defining sets for calcium signaling pathway genes, F-actin dynamics genes, pro-7-ketocholesterogenic genes and migration-associated genes described in **Supplementary Table 4** were scored by using the Seurat’s AddModuleScore function. After T cells were stratified into high-and low-expression groups of target gene sets or target genes by the median of scores or expression levels, the migration-associated gene set scores of the groups were compared. Functional enrichment analysis was conducted using Gene Ontology (GO) terms and the KEGG pathways retrieved from authoritative databases including NCBI, UniProt, and KEGG official resources, aiming to elucidate key pathways and biological processes.

### 2.6 Intracellular Ca^2+^ concentration detection

To detect intracellular free Ca^2+^ levels, Fluo-3 AM (S1056, Beyotime, China), a calcium-sensitive fluorescent probe, was applied. Briefly, cells were loaded with 5 μM Fluo-3 AM at 37°C for 1 h, followed by PBS wash for 3 times. To ensure complete hydrolysis of Fluo-3 AM into its Ca^2+^-sensitive form (Fluo-3), cells were further incubated at 37°C for 30 min. Subsequently, the cells were washed and resuspended in 300 μL PBS for flow cytometry, and the signal was detected at 488 nm excitation and 525 nm emission.

### 2.7 Detection of F-actin

F-actin in cells was labeled with Actin-Tracker Green-488 (C2201S, Beyotime, China), a green-fluorescent probe conjugated to Alexa Fluor 488-phalloidin. Briefly, harvested cells were washed with PBS, suspended on glass slides, and air-dried at room temperature (RT). The cells were then fixed with 3.7% formaldehyde in PBS for 20 min at RT, followed by three 5-min washes with PBS containing 0.1% Triton X-100. For staining, Actin-Tracker Green was diluted to a working solution (1:100) in PBS containing 3% BSA and 0.1% Triton X-100. A 200 µL aliquot of the working solution was applied to each slide, followed by incubation at RT for 60 min in the dark. After incubation, slides were washed 2–4 times (5 min each) with PBS containing 0.1% Triton X-100. Cell nuclei were counterstained with antifade mounting medium containing DAPI/PI (Beyotime, China). Fluorescence signals were visualized using a fluorescence microscope (Leica, Germany) and a Stimulated Emission Depletion (STED) confocal microscope (Abberior, Stedcyon, Germany).

### 2.8 Immunocytofluorescence

Cells were PBS-washed, resuspended, and plated on slides for air-drying at room temperature. Fixed with 4% paraformaldehyde (30 min) and permeabilized with 0.5% Triton X-100 (20 min; Servicebio, China), samples were blocked with 3% BSA (37 °C, 1 h; Servicebio, China). Immunostaining included overnight incubation at 4 °C with anti-NFATc1 (1:200; Proteintech) followed by Alexa Fluor^®^ 488-secondary antibody (1:500; Proteintech, 1 h, RT). Antifade mounting medium with DAPI (Beyotime, China) was applied to counterstain nuclei. Imaging used both fluorescence microscope (Leica, Germany) and STED confocal microscope (Abberior, Stedcyon, Germany).

### 2.9 Cytotoxity assay

The cytotoxic effects of 7KC at various concentrations and treatment durations were assessed using the Enhanced Cell Counting Kit-8 (CCK8, E-CK-A362, elabscience, Wuhan, China) according to the manufacturer’s protocol. Briefly, cells were seeded in 96-well plates at a density of 5 × 10^4^ cells/well in 100 μL complete medium containing varying concentrations of 7KC or dimethyl sulfoxide (DMSO). At the desired time point for each group, 10 μL of CCK-8 reagent was added to each well, followed by incubation for 2.5 h at 37 °C. Wells containing culture medium plus CCK-8 without any cells served as the blank control. The optical density (OD) was measured at 450 nm using a 96-well multiscanner autoreader (Thermo Fisher Scientifc).

### 2.10 Cell culture and transwell co-culture

Primary T cells, and Jurkat T cells were maintained in RPMI 1640 medium (HyClone, USA) containing 10% fetal bovine serum at a density of 1 × 10^6^ cells/mL under standard conditions (37°C, 5% CO_2_). T cell activation was induced by co-stimulation with 2 µg/mL anti-CD28 mAb (Biolegend, USA) and 1 µg/mL anti-CD3 mAb (Biolegend, USA) for 72 h. To simulate the peripheral immune microenvironment, activated Jurkat T cells were cultured in neat plasma (without mixing or dilution in culture medium) from OLP patients or healthy controls at density of 1 × 10^6^ cells/mL for 48 h (37°C, 5% CO_2_). For investigating the immunomodulatory effects of 7KC, activated primary T cells and plasma-treated Jurkat T cells were exposed to 5 μM 7KC in fresh medium for 24 h (37°C, 5% CO_2_). To examine calcium signaling involvement, further cultures were treated with 7KC followed by a 1-hour incubation with the calcium channel blocker CM4620 (MedChemExpress, USA). Both 7KC and CM4620 were prepared in DMSO, with DMSO at a concentration of 0.1% (v/v) serving as negative solvent controls.

Human oral keratinocytes (HOK cell line, six-passage subculture) were plated at 1 × 10^5^ cells/well in 24-well plates using KGM-Gold: DMEM (2:1, Lonza: Servicebio) medium. After 24 h incubation (37°C), cells were LPS-treated (10 μg/mL, Solarbio) for 24 h, PBS-washed twice, then prepared for co-culture.

An indirect co-culture system was established in 5 μm pore transwell plates (24-well format) using LPS-pretreated keratinocytes in the lower chamber (500 μL of 2:1 KGM-Gold: DMEM medium) and pre-activated primary T cells/Jurkat T cells (Jurkat T cells: 5 × 10^5^; primary T cells: 2 × 10^6^ cells in 200 μL RPMI-1640) which were incubated with 7KC or/and CM4620 (supplemented at 8-hour intervals) as described above in the upper chamber. After incubation at 37°C for 24 h, migrated cells in the lower chamber were quantified and imaged.

### 2.11 Quantitative real-time polymeras chain reaction

Primary T cell RNA was extracted with the FastPure Cell/Tissue Total RNA Isolation Kit V2 (Vazyme, China). RNA quality was verified by measuring absorbance ratios (260/280 nm) using a NanoDrop 2000 spectrometer (Thermo Fisher Scientific, USA). For cDNA synthesis, extracted RNA was reverse transcribed using the PrimeScript™ RT reagent kit with integrated gDNA removal (Takara Bio, China). Quantitative PCR was performed on an ABI 7500 system (Applied Biosystems, USA) with 2×Universal SYBR Green Fast qPCR Mix (Abclonal, China). Gene-specific primers (Sangon Biotech, China; sequences listed in **Supplementary Table 5**) were applied. Relative gene expression was calculated by the comparative Ct method (2^−ΔΔCt^), with GAPDH serving as the endogenous control.

### 2.12 Cell apoptosis assay

Keratinocyte apoptosis was assessed with an Annexin V-APC/PI apoptosis assay kit (KeyGEN, China). Briefly, keratinocytes were detached using a trypsin solution (EDTA-free; Beyotime, China) and washed twice with PBS. The cells were then resuspended in binding buffer at a concentration of 1 × 10^6^ cells/mL and incubated with FITC-labeled Annexin V and PI for 15 minutes at room temperature, protected from light. Apoptotic cells were quantified via flow cytometry (Beckman Coulter) within 1 h post-staining. Data analysis was performed using CytExpert software, with apoptosis rates calculated as the proportion of Annexin V-positive/PI-negative cells.

### 2.13 Statistical analysis

Data analysis was conducted using GraphPad Prism 8.0 (GraphPad Software, San Diego, CA, USA). The normality of data distribution was evaluated using the Kolmogorov-Smirnov test, while variance homogeneity was verified by the F-test. For comparisons between independent groups, either an unpaired Student’s t-test (for normally distributed data with equal variances) or one-way ANOVA followed by Tukey’s multiple comparison test was employed. When variances were unequal, Welch’s correction was applied. Paired samples were analyzed using a paired Student’s t-test. Results are presented as mean ± SEM, with statistical significance set at p < 0.05. Statistical power > 0.8 for all outcomes was ensured by PASS calculations. Heatmaps were generated after Z-score normalization of raw values.

## 3 Results

### 3.1 Abnormal steroids in OLP and their functions

**Figure 1:**
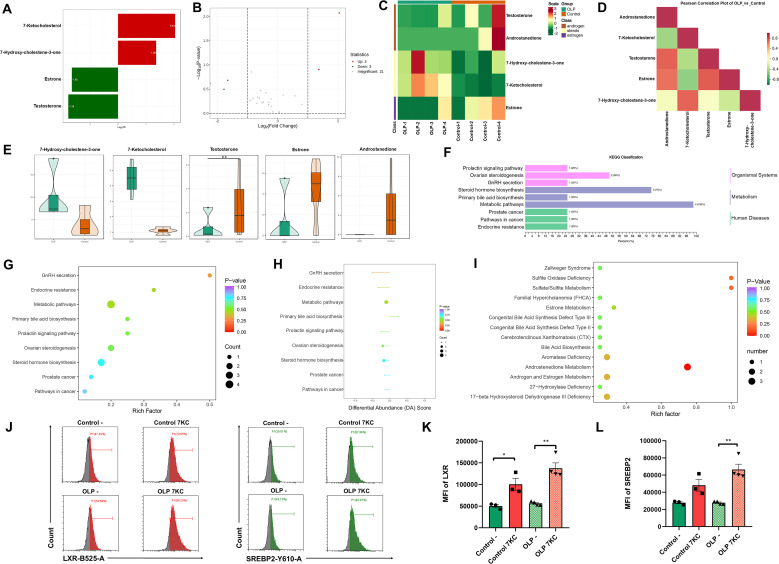
Abnormal steroids in OLP plasma and their functions. **(A–E)**. Steroids in plasma from healthy donors (n=4) and OLP patients (n=4) were determined by LC-MS/MS, and the results of the top 20 metabolites by differential folds were shown as bar chart **(A)**; The results of the top 10 metabolites with the highest FC value were shown in the Volcano diagram **(B)**; Clustering heat map **(C)**; The heat map of correlation between differential metabolites **(D)**; The violin diagram **(E)** of differential metabolites. **(F–I)**. Functional annotation and enrichment analysis of differential metabolites were performed. The bar chart of KEGG classification with the proportion of differential metabolites assigned to each pathway **(F)**, the bubble plots of KEGG functional enrichment **(G)**, differential abundance scores **(H)** and HMDB enrichment analysis **(I)**. **(J–L)**. Flow cytometry was used to determine the levels of cellular cholesterol metabolism regulatory axis SREBP2-LXR in primary T cells treated/untreated with 7-ketocholesterol **(J)**, and the mean fluorescence intensity (MFI) of LXR **(K)** and SREBP2 **(L)** were calculated. Data were presented as mean ± SEM. *p < 0.05, **p < 0.01, 7KC, 7-Ketocholesterol.

Given that dysregulated cholesterol in OLP is the central precursor molecule for all steroid metabolites, which modulate T cell responses in autoimmunity, we aimed to identify the dominant disrupted steroids in the plasma of OLP patients. Plasma concentrations of 7KC in OLP patients were significantly higher than in healthy controls, with its positively correlated metabolite 7-hydroxycholestene-3-one exhibiting an upward trend (**Figures 1A–E**). Conversely, the levels of positively associated testosterone, estrone, and androstenedione exhibited a downward trend (**Figures 1A–E**). KEGG classification of metabolites with abundance changes in OLP highlighted their involvement in steroid hormone biosynthesis and ovarian steroidogenesis (**Figure 1F**). No pathways that reached the statistically significant threshold were found in the KEGG pathway enrichment and differential abundance score analysis (**Figures 1G, H**). HMDB functional enrichment analysis showed that differential metabolites were significantly enriched in “androstenedione metabolism” functional category (**Figure 1I**). HMDB database annotation identified 7KC as a metabolite previously implicated in the pathogenesis of the autoimmune disease (**Supplementary Table 6** (10, 34, 35). Further investigation using flow cytometry demonstrated that 7KC exerted a counter-regulatory effect on the cholesterol metabolic axis SREBP2-LXR in OLP T cells, with significant upregulation of both SREBP2 and LXR expression (**Figures 1J–L**).

### 3.2 ScRNA-seq revealed dysregulated pro-7-ketocholesterogenic genes and a hyperactive Ca^2+^-NFATc1-F-actin/LFA-1α signaling axis linked to T cell migration in local OLP T cells

**Figure 2:**
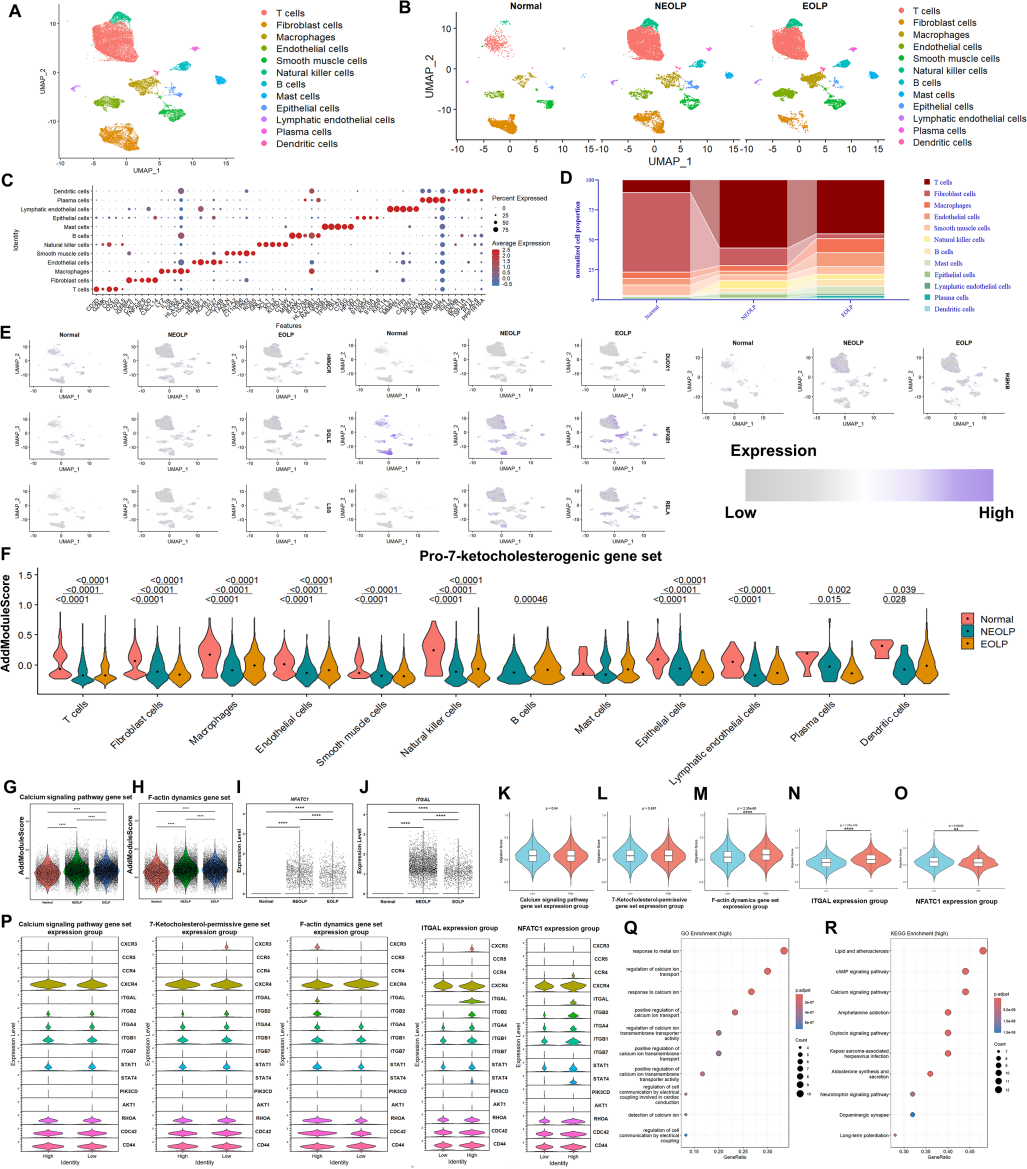
ScRNA-seq revealed dysregulated pro-7-ketocholesterogenic genes and a hyperactive Ca^2+^-NFATc1-F-actin/LFA-1α signaling axis linked to T cell migration in local OLP T cells. **(A, B)** The UMAP for all cell types **(A)**, and cell origins in normal, NEOLP and EOLP subjects **(B)**. **(C)** The dot plot showing the expression of marker genes for each cell type. **(D)** The proportion of each cell type in OLP and normal samples. **(E)**. The UMAP plots showing the expressions of pro-7-ketocholesterogenic genes in each cell type from NEOLP, EOLP and normal samples. **(F)**. Scores of pro-7-ketocholesterogenic gene set in various types of cells in NEOLP, EOLP and normal samples. **(G–J)** The scores of calcium signaling pathway gene set **(G)** and F-actin dynamics gene set **(H)**, and the expression levels of *NFATC1***(I)** and *ITGAL***(J)** in T cells of normal, NEOLP and EOLP samples. **(K–P)** The violin plots showing the different scores of migration-associated gene set between calcium signaling pathway gene set-high and calcium signaling pathway gene set-low T cells **(K)**, pro-7-ketocholesterogenic gene set-high and pro-7-ketocholesterogenic gene set-low T cells **(L)**, F-actin dynamics gene set-high and F-actin dynamics gene set-low T cells **(M)**, *ITGAL*-high and *ITGAL*-low T cells **(N)**, *NFATC1*-high and *NFATC1*-low T cells **(O)** in OLP and control tissues. The levels of specific migration-related genes in T cells of these groups were also shown **(P)**. **(Q, R)** The bubble plots showed GO enrichment analysis **(Q)** and KEGG pathway analysis **(R)** of differentially expressed genes (DEGs) between calcium signaling pathway gene set-high and calcium signaling pathway gene set-low T cells groups. **p < 0.01, ****P < 0.0001.

Visualization using UMAP revealed cell clusters that were annotated as 12 major cell types from normal, NEOLP, EOLP samples (**Figures 2A, B**), based on the marker gene expression (**Figure 2C**). Compared with normal tissues, OLP samples exhibited a more pronounced increase in T cell proportion relative to other cell types, followed by elevated ratios of NK cells, B cells, and mast cells in OLP, increased epithelial cells in NEOLP, and higher proportions of macrophages, endothelial cells, lymphatic endothelial cells, plasma cells, and dendritic cells in EOLP (**Figure 2D**). UMAP visualization of pro-7-ketocholesterogenic genes which function in substrate production (e.g., *HMGCR*, *SQLE*, *LSS*, *CYP7A1*), enzymatic synthesis (e.g., *ALOX15*, *ALOX12*), and oxidative stress generation (e.g., *NOX1-5*, *DUOX1/2*, *NFKB1*, *RELA*, *IKBKB*, *HIF1A*) across NEOLP, EOLP, and normal tissue cell clusters revealed expression of the substrate biosynthesis genes (*HMGCR*, *SQLE*, *LSS*) and the pro-oxidative genes (*RELA*, *DUOX1*) in multiple clusters, including T cells, with particularly increased levels of *RELA* and *SQLE* (**Figure 2E**). However, the aggregate scoring of the pro-7-ketocholesterogenic gene set revealed lower scores of most cells in OLP than the normal counterparts, including T cells, fibroblasts, macrophages, endothelial cells, smooth muscle cells, natural killer cells, epithelial cells, lymphatic endothelial cells, plasma cells, and dendritic cells (**Figure 2F**). Building on the role of 7KC in activating Ca^2+^-NFATc1 pathway, and the downstream F-actin/LFA-1α-mediated pathogenic effects in OLP, the Ca^2+^-NFATc1-F-actin/LFA-1α axis in local OLP T cells was further explored (12, 28, 29). The scores of calcium signaling pathway gene set (**Figure 2G**), F-actin dynamics gene set (**Figure 2H**), *NFATC1* expression levels (**Figure 2I**), and *ITGAL* (the gene encoding LFA-1α) (**Figure 2J**) in local OLP T cells were significantly higher than those in the control group, with the highest levels observed in NEOLP, indicating Ca^2+^-NFATc1-F-actin/LFA-1α signaling axis was active in local OLP T cells. Furthermore, T cells with high level of the F-actin dynamics gene set (**Figure 2M**) or *ITGAL* (**Figure 2N**) exhibited significantly elevated AddModuleScores for migration-associated genes. In contrast, *NFATC1*-high T cells showed reduced migration-associated gene scores (**Figure 2O**). No significant differences were observed between high- and low-expression T cell groups for the calcium signaling pathway (**Figure 2K**) or pro-7-ketocholesterogenic gene set (**Figure 2L**). Violin plots of migration-associated genes revealed upregulations of chemokine gene *CXCR3*, adhesion-related genes *ITGAL*/*ITGB2*, and cytoskeletal regulatory genes *RHOA*/*CDC42* in F-actin dynamics gene set-high T cells, elevated expressions of chemokine gene *CXCR3*, adhesion-related genes *ITGB2*/*ITGA4*/*ITGB1*, and transcriptional activator *STAT4* in *ITGAL*-high T cells, and increased levels of chemokine genes *CCR4*/*CXCR4*, adhesion-related genes *ITGAL*/*ITGB2*, and transcriptional activators *STAT1*/*STAT4* in *NFATC1*-high T cells (**Figure 2P**). GO analysis revealed that T cells with high calcium signaling pathway gene set scores showed significant enrichment in “Regulation of cell communication by electrical coupling” and calcium ion detection, transport regulation, transmembrane activity modulation, and downstream cellular responses (**Figure 2Q**). KEGG analysis revealed calcium signaling-high T cells in OLP were linked to diverse pathways including “Lipid and atherosclerosis”, “cAMP signaling pathway”, “Calcium signaling pathway”, “Amphetamine addiction”, “Oxytocin signaling pathway”, “Aldosterone synthesis and secretion”, “Neurotrophin signaling pathway”, “Dopaminergic synapse” and “Long-term potentiation” (**Figure 2R**).

### 3.3 The Ca^2+^-NFATc1-F-actin/LFA-1α signaling axis was significantly activated in peripheral OLP T cells

**Figure 3:**
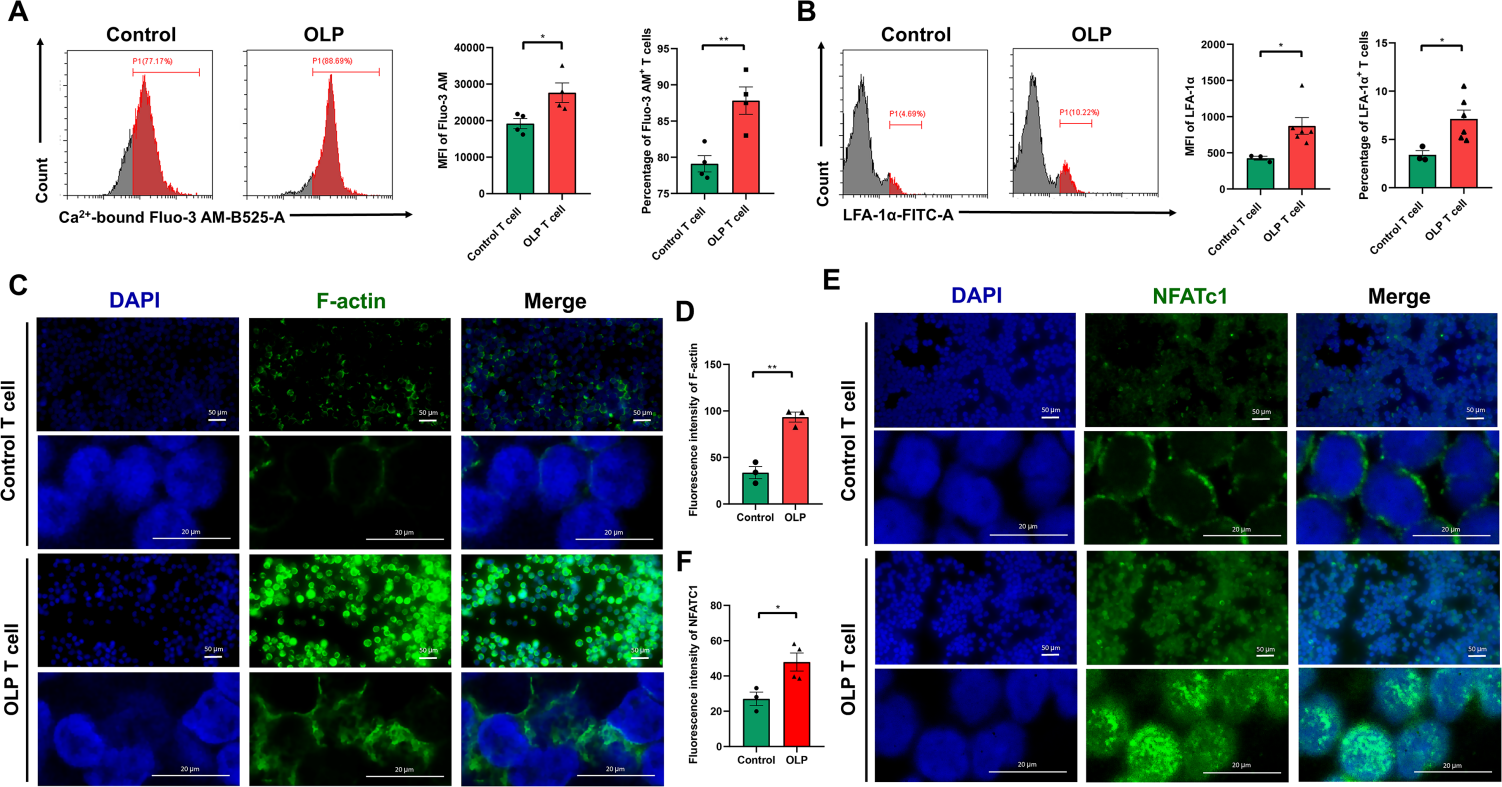
The Ca^2+^-NFATc1-F-actin/LFA-1α signaling axis was significantly activated in peripheral OLP T cells. **(A, B)** Flow cytometry was used to determine the levels of Ca^2+^-bound-Fluo-3 AM **(A)** and LFA-1α **(B)** in primary control/OLP T cells, with the MFI of Ca^2+^-bound Fluo-3 AM and LFA-1α, and the percentage of Ca^2+^-bound Fluo-3 AM^+^ T cells and LFA-1α^+^ T cells calculated. **(C-F)** Representative images of immunocytofluorescent staining for F-actin **(C)** and NFATc1 **(E)** in primary control/OLP T cells by fluorescence microscope (Scale bars, 50 μm) and confocal microscope (Scale bars, 20 μm). The fluorescence intensity of total F-actin **(D)** and nuclear NFATc1 **(F)** was measured. Data were presented as mean ± SEM. *p < 0.05, **p < 0.01.

To investigate the Ca^2+^-NFATc1-F-actin/LFA-1α signaling axis in peripheral OLP T cells, PBMCs from OLP patients were isolated, and CD3^+^ T cells were purified. Flow cytometry analysis revealed significantly elevated levels of intracellular Ca^2+^ (**Figure 3A**) and LFA-1α (**Figure 3B**) in OLP T cells compared to controls. Fluorescent probe labeling of F-actin in purified OLP CD3^+^ T cells demonstrated enhanced F-actin expression and polymerization, with confocal microscopy revealing denser and more elongated filamentous structures (**Figure 3C**), and statistical analysis confirmed significant upregulation of F-actin (**Figure 3D**). Immunofluorescence staining revealed differential subcellular localization of NFATc1, showing predominantly cytoplasmic distribution in control T cells, with marked nuclear staining in OLP T cells (**Figure 3E**), and statistical analysis confirmed markedly increased nuclear NFATc1 expression in OLP T cells compared to controls (**Figure 3F**).

### 3.4 7-Ketocholesterol activated the Ca^2+^-NFATc1-F-actin/LFA-1α signaling axis in OLP T cells

**Figure 4:**
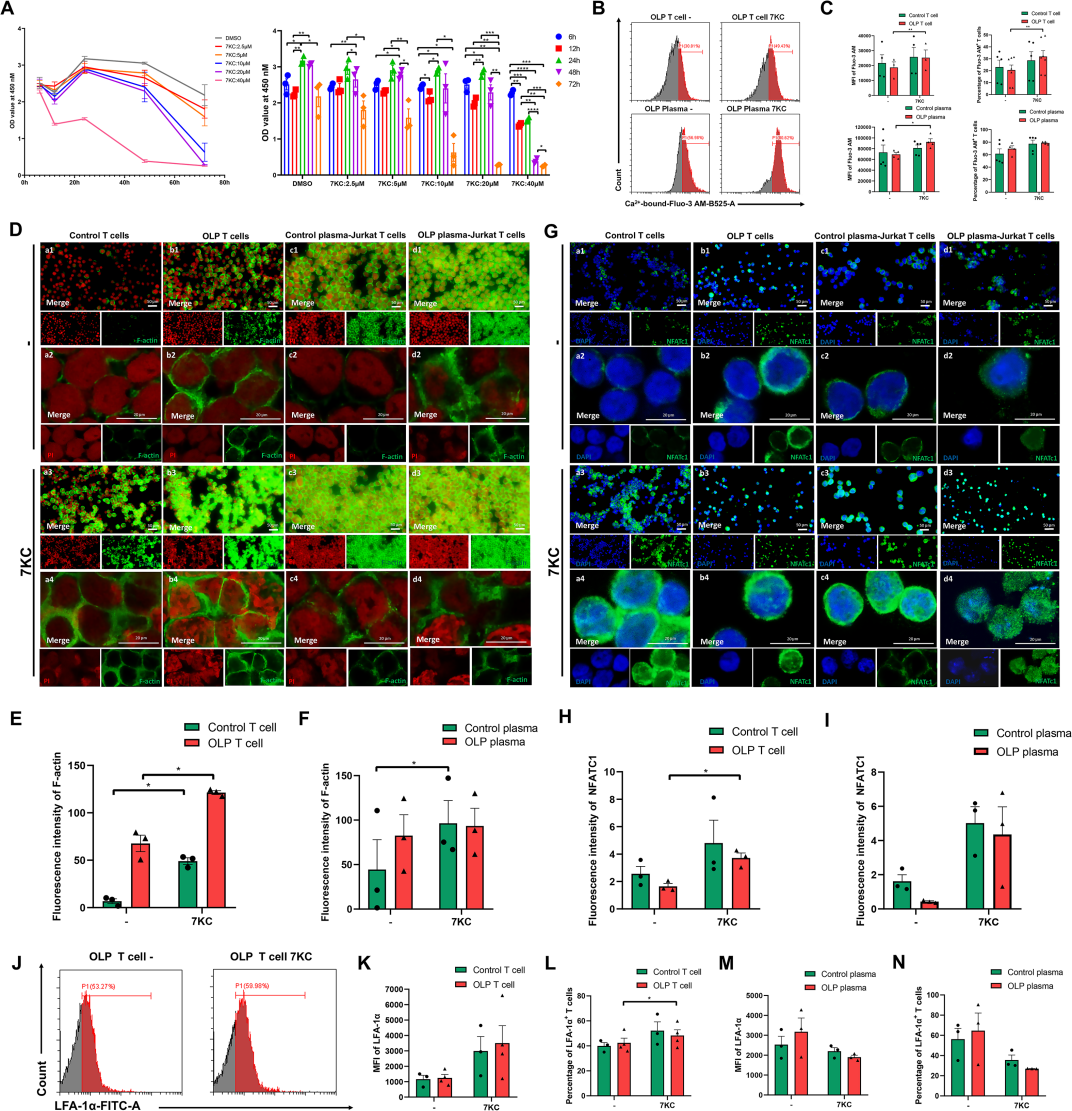
7-Ketocholesterol activated the Ca^2+^-NFATc1-F-actin/LFA-1α signaling axis in OLP T cells. Primary control/OLP T cells and Jurkat T cells were activated by 1 µg/mL anti-CD3 mAb and 2 µg/mL anti-CD28 mAb for 72 h For activated Jurkat T cells, control/OLP plasma then replaced the medium for 2 days. **(A)** Activated Jurkat T cells were seeded in a 96-well plate and treated with different doses of 7-ketocholesterol (2.5 µM, 5 µM, 10 µM, 20 µM, 40 µM) for 6 h, 12 h, 24 h, 48 h or 72 h Cytotoxity was determined by the OD value at 450 nm using the CCK8 assay kit. **(B, C)** Flow cytometry was used to determine the levels of Ca^2+^-bound-Fluo-3 AM after 5 µM 7-ketocholesterol treatment for 24 h Representative image of Ca^2+^-bound Fluo-3 AM levels of primary OLP T cells and OLP plasma-pretreated Jurkat T with or without 7-ketocholesterol treatment **(B)**, and the mean fluorescence intensity (MFI) and the percentage of Ca^2+^-bound Fluo-3 AM^+^ T cells were calculated **(C)**. **(D–I)** Representative images of immunocytofluorescent staining for F-actin expression **(D)** and NFATc1 **(G)** in control/OLP T cells and control/OLP plasma-treated Jurkat T cells after 5 µM 7-ketocholesterol treatment for 24 h by fluorescence microscope (Scale bars, 50 μm) and confocal microscope (Scale bars, 20 μm). The fluorescence intensity of F-actin in primary T cells **(E)** and Jurkat T cells **(F)**, and nuclear NFATc1 in primary T cells **(H)** and Jurkat T cells **(I)** were measured. J-N. After cells were incubated with 5 µM 7-ketocholesterol for 24 h, the expression levels of LFA-1α was detected via flow cytometry **(J)**, and the MFI of LFA-1α in primary T cell **(K)** and Jurkat T cells **(M)**, and the percentage of LFA-1α^+^ primary T cells **(L)** and LFA-1α^+^ Jurkat T cells **(N)** were calculated. DMSO at a concentration of 0.1% (v/v) was used as the solvent control. Data were presented as mean ± SEM. *p < 0.05, **p < 0.01, ***p < 0.001, ****p < 0.0001, 7KC, 7-Ketocholesterol.

Aiming at delineating the mechanistic impact of 7KC on the Ca^2+^-NFATc1-F-actin/LFA-1α axis in OLP T cells, we first established 7KC treatment conditions (5 μM, 24 h; **Figure 4A**). Using Fluo-3 AM probes, flow cytometry revealed that 7KC significantly increased the mean fluorescence intensity (MFI) of Ca^2+^-bound Fluo-3 AM in primary OLP T cells, and the percentage of Ca^2+^-bound Fluo-3 AM^+^ primary OLP T cells (**Figures 4B, C**). The Jurkat T cells were treated with OLP plasma mimicking the OLP peripheral microenvironment, and the MFI of Ca^2+^-bound Fluo-3 AM in OLP plasma-treated Jurkat T cells was also significantly elevated by 7KC (**Figures 4B, C**). Expression and polymerization of F-actin in primary control/OLP T cells and control plasma-treated Jurkat T cells were enhanced after incubation with 7KC (**Figures 4D–F**). Immunofluorescence demonstrated significantly potentiated 7KC-induced NFATc1 nuclear expression in primary OLP T cells, with upregulated trends in primary control T cells and control/OLP plasma-treated Jurkat T (**Figures 4G–I**). Flow cytometry showed that 7KC significantly elevated the LFA-1α level in primary OLP T cells, but showed a downregulation trend in control/OLP plasma-treated Jurkat T cells (**Figures 4J–N**).

### 3.5 7-Ketocholesterol enhanced inflammatory profile and migration of OLP T cells

**Figure 5:**
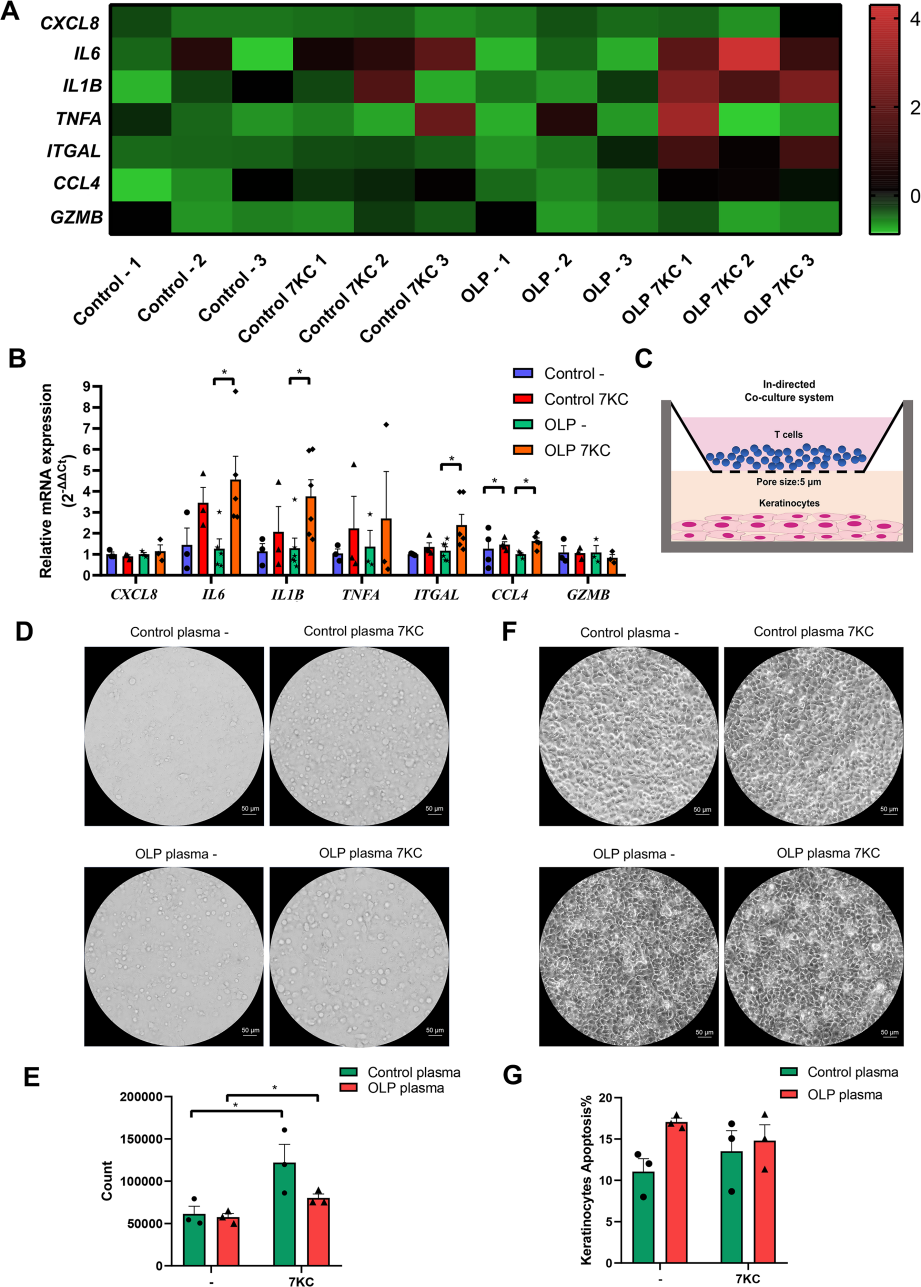
Inflammatory cytokine profiles and migration capacity of OLP T cells were augmented by 7-ketocholesterol treatment. **(A, B)** Activated primary control/OLP T cells were treated with 5 µM 7-ketocholesterol for 24 h The levels of cytokine genes were measured by qRT-PCR. The heat map showed the relative expression of genes **(A)** and the histogram showed the exact statistical significance **(B)**. **(C–G)**. Activated control/OLP plasma-treated Jurkat T cells, which were treated with 5 µM 7-ketocholesterol for 24 h, were co-cultured with LPS-pretreated (10 μg/mL, 24 h) keratinocytes for 24 h in the indirect co-culture system. Graphic representation of the indirect co-culture model system was shown **(C)**. Cells that migrated to the lower chamber were photographed **(D)** (Scale bars, 50 μm) and quantified **(E)**. Keratinocytes in the lower chambers were photographed after T cells were harvested **(F)** (Scale bars, 50 μm), and the apoptosis of them was measured by flow cytometry and calculated by the percentage of Annexin V^+^/PI^−^ cells **(G)**. DMSO at a concentration of 0.1% (v/v) was used as the solvent control. Data were presented as mean ± SEM. *p < 0.05, 7KC, 7-Ketocholesterol.

The effects of 7KC on the migratory and cytotoxic inflammatory molecules in primary OLP T cells were further explored via qRT-PCR, and the result showed that the mRNA levels of *IL6*, *IL1B* and *ITGAL* in primary OLP T cells, and *CCL4* (encoding MIP-1β) in primary control/OLP T cells were significantly elevated after 7KC treatment, while *CXCL8*, *TNFA*, and *GZMB* levels remained unchanged (**Figures 5A, B**). An indirect transwell co-culture model of control/OLP plasma-pretreated Jurkat T cells and LPS-pretreated keratinocytes (to mimic localized inflammation) was established (**Figure 5C**). Photographing and counting of T cells migrating to the lower chamber of the transwell co-culture systems showed a pronounced increase in the number of control/OLP plasma-pretreated Jurkat T cells incubated with 7KC (**Figures 5D, E**). Flow cytometry showed that 7KC treatment did not affect the cytotoxic effect of T cells on keratinocytes (**Figures 5F, G**).

### 3.6 Calcium channel blockade suppressed F-actin and nuclear NFATc1 expression in 7-ketocholesterol-pretreated OLP T cells

**Figure 6:**
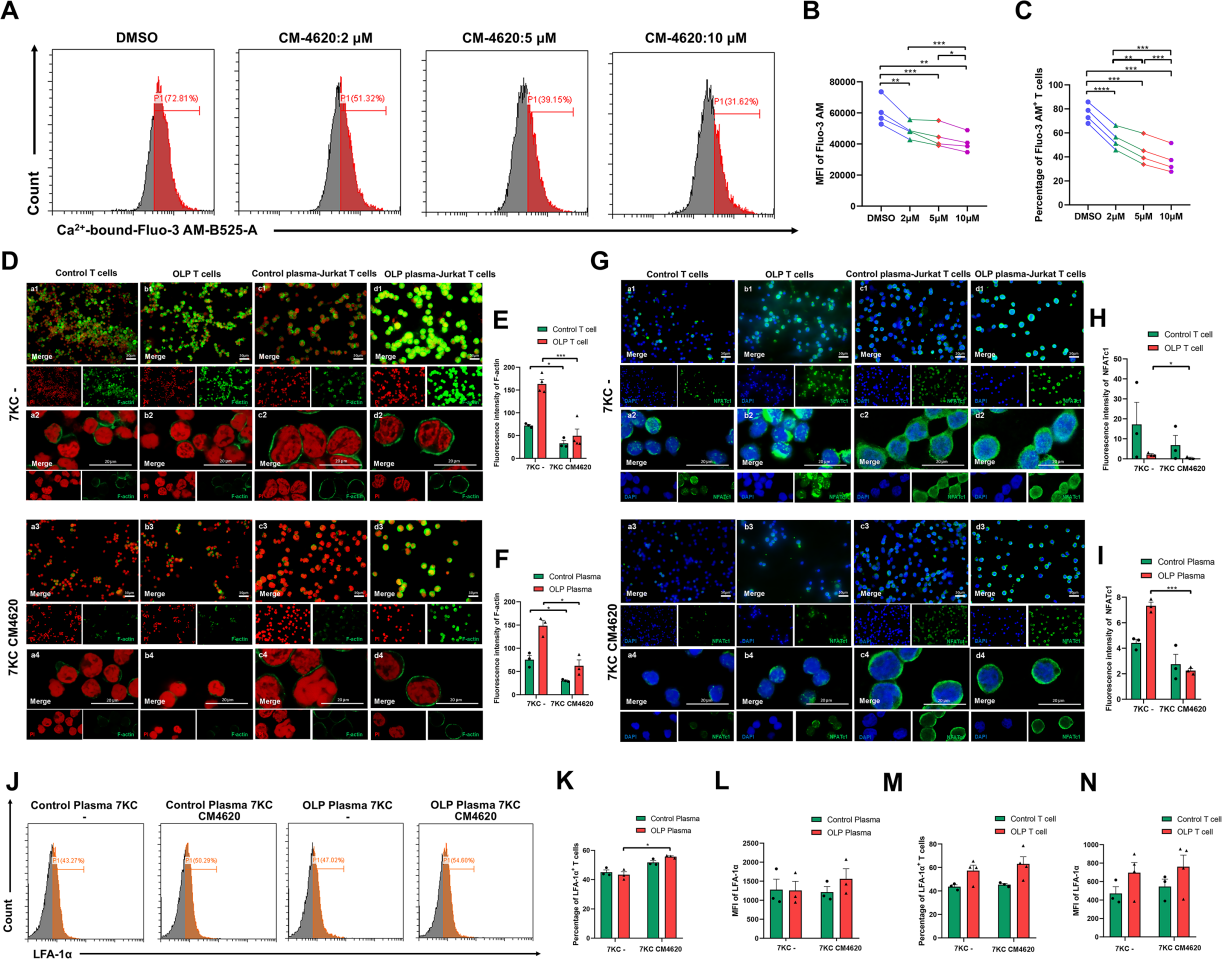
Calcium channel blockade suppressed F-actin and nuclear NFATc1 expression in 7-ketocholesterol-pretreated OLP T cells. **(A–C)** Activated Jurkat T cells were treated with different doses of the calcium channel-blocking agent CM4620 (2 µM, 5 µM, 10 µM) for 1 h Flow cytometry was used to determine the levels of Ca^2+^-bound Fluo-3 AM in Jurkat T cells **(A)**, and the MFI of Ca^2+^-bound Fluo-3 AM **(B)** and the percentage of Ca^2+^-bound Fluo-3 AM^+^ T cells **(C)** were calculated. **(D–N)** After priming with 5 μM 7-ketocholesterol for 24 h, activated primary control/OLP T cells and control/OLP plasma-treated Jurkat T cells were exposed to 10 µM CM4620 for 1 **(h)** Representative images of immunocytofluorescent staining for F-actin expression **(D)** and NFATc1 **(G)** in control/OLP T cells and control/OLP plasma-treated Jurkat T cells by fluorescence microscope (Scale bars, 50 μm) and confocal microscope (Scale bars, 20 μm). The fluorescence intensity of F-actin in primary T cells **(E)** and Jurkat T cells **(F)**, and NFATc1 in primary T cells **(H)** and Jurkat T cells **(I)** were measured. The expression levels of LFA-1α was detected via flow cytometry **(J)**, and the percentages of LFA-1α^+^ Jurkat T cells **(K)** and LFA-1α^+^ primary T cells **(M)** were calculated, along with the MFI of LFA-1α in Jurkat T cells **(L)** and primary T cell **(N)**. DMSO at a concentration of 0.1% (v/v) was used as the solvent control. Data were presented as mean ± SEM. *p < 0.05, **p < 0.01, ***p < 0.001, ****p < 0.0001, 7KC, 7-Ketocholesterol.

In order to elucidate whether 7KC is dependent on the Ca^2+^-NFATc1 signaling pathway to promote the expressions of LFA-1α and F-actin, and F-actin assembly in OLP T cells, the Ca^2+^ channel blocker CM4620 was employed. Flow cytometry identified 10 μM CM4620 as the optimal concentration for effective Ca^2+^ channel blockade (**Figures 6A–C**). Ca^2+^ channel inhibition significantly attenuated F-actin expression in 7KC-pretreated primary control/OLP T cells and plasma-pretreated Jurkat T cells (**Figures 6D–F**). Immunofluorescence revealed that the blockade of Ca^2+^ channel abolished nuclear NFATc1 in 7KC-primed primary OLP T cells and OLP plasma-pretreated Jurkat T cells, with a downward trend in primary control T cells and control plasma-treated Jurkat T cells (**Figures 6G–I**). While Ca^2+^ channel blockade did not significantly alter LFA-1α expression in 7KC-pretreated primary T cells, it paradoxically upregulated LFA-1α in OLP plasma-treated Jurkat T cells (**Figures 6J–N**).

### 3.7 Proinflammatory cytokine profiles, F-actin assembly and cell migration were attenuated by calcium channel inhibition in 7-ketocholesterol-pretreated OLP T cells

**Figure 7:**
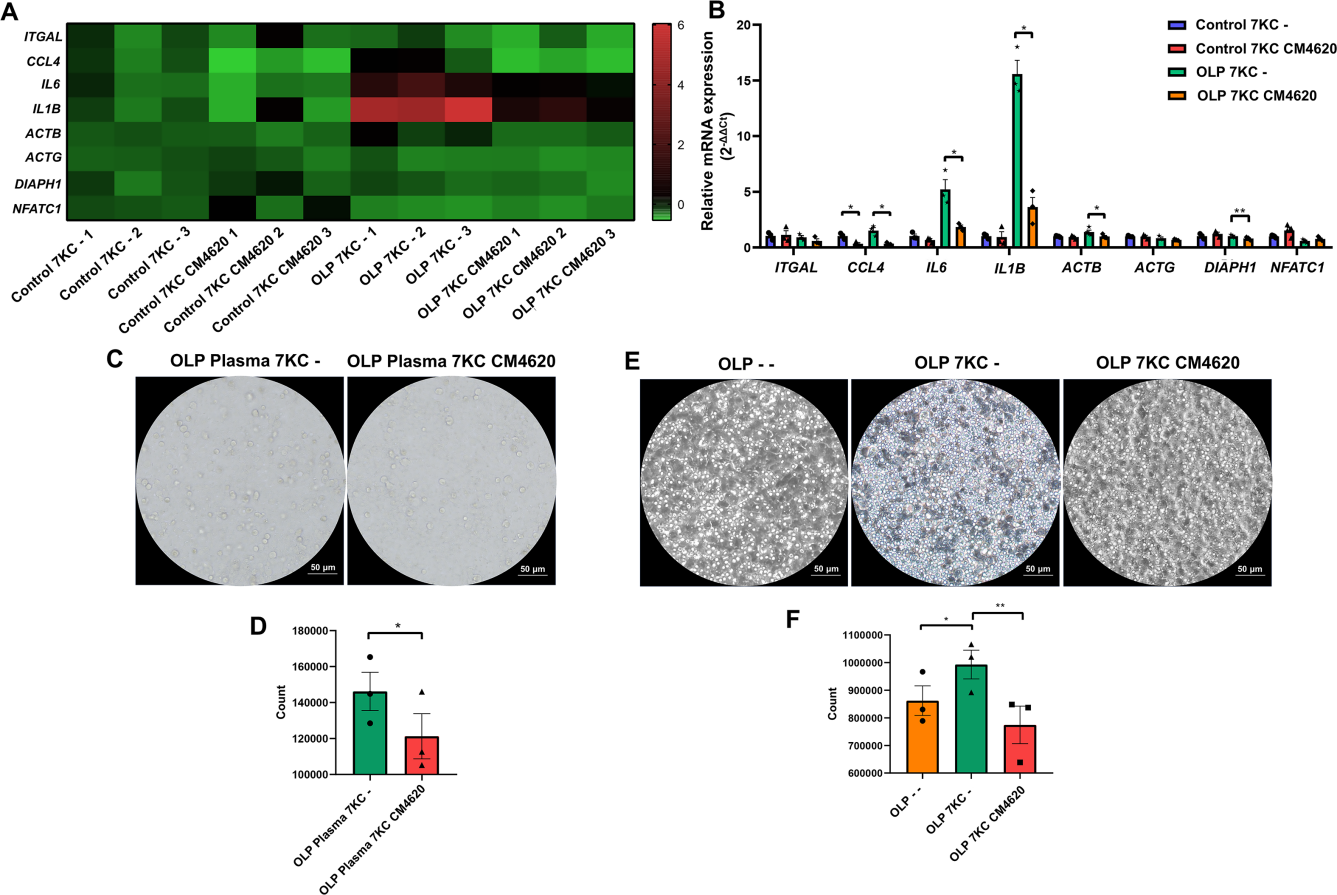
Proinflammatory cytokine profiles, F-actin assembly, and cell migration were attenuated by calcium channel inhibition in 7-ketocholesterol-pretreated OLP T cells. **(A, B)** Activated primary control/OLP T cells were pretreated with 5 µM 7-ketocholesterol for 24 h, and then incubated with 10 µM CM4620 for 1 h The levels of cytokine genes and F-actin assembly-related genes were measured by qRT-PCR. The heat map showed the relative expression of genes **(A)** and the histogram showed the exact statistical significance **(B)**. **(C–F)** After 5 μM 7-ketacholesterol treated primary OLP T cells or OLP plasma-treated Jurkat T cells for 24 h, 10 µM CM4620 was applied for 1 h, and these T cells were then co-cultured with LPS-pretreated (10 μg/mL, 24 h) keratinocytes for 24 h in the indirect co-culture system. OLP plasma-treated Jurkat T cells **(C)** and primary OLP T cells **(E)** that migrated to the lower chamber were photographed (Scale bars, 50 μm) and quantified [Jurkat T cells: **(D)**; primary T cells: **(F)**]. DMSO at a concentration of 0.1% (v/v) was used as the solvent control. Data were presented as mean ± SEM. *p < 0.05, **p < 0.01, 7KC, 7-Ketocholesterol.

qRT-PCR analysis revealed that calcium pathway blockade significantly suppressed mRNA levels of *IL6* and *IL1B* in primary OLP T cells, *CCL4* in primary control/OLP T cells, and F-actin polymerization-related genes *ACTB* and *DIAPH1* (encoding mDia) in primary OLP T cells (**Figures 7A, B**). In transwell co-culture assays with LPS-primed keratinocytes, Ca^2+^ channel inhibition markedly reduced the number of 7KC-pretreated and OLP plasma-conditioned Jurkat T cells migrating to the lower chamber (**Figures 7C, D**). The migration of 7KC-treated primary OLP T cells in transwell co-culture system was enhanced, but attenuated after Ca^2+^ channel blockade (**Figures 7E, F**).

## 4 Discussion

Oral lichen planus (OLP), a chronic T-cell-mediated inflammatory disorder of unclear etiology with female predominance, is increasingly associated with dysmetabolic features, including elevated plasma cholesterol/low-density lipoprotein (LDL) and localized oxidative stress (1, 6, 8). As downstream products of cholesterol oxidation, oxysterols are now recognized as immunomodulators in autoimmune diseases such as systemic lupus erythematosus (SLE) and MS, for promoting pathogenic T cell migration and activation (7, 36). The oxLDL-derived oxysterol 7KC was upregulated across chronic inflammatory conditions, such as psoriasis, IBD and MS, positively correlated with the disease severity of MS, and aggravated the inflammatory cell infiltration and cytokine secretion in psoriasis (10). In this study, LC-MS/MS profiling of OLP plasma revealed abnormalities in cholesterol metabolite steroid patterns, with marked elevation of 7KC and a concomitant increase in its metabolic precursor 7-hydroxy-cholest-4-en-3-one. Conversely, androstenedione, testosterone, and estrone, which were positively correlated with each other, showed a downward trend, consistent with the endocrine shifts characteristic of menopausal women with OLP predisposition (37). Significant enrichment of OLP-altered metabolites in “androstenedione metabolism” points to disturbed sex hormone homeostasis, aligning the clinical link to phases of hormonal instability like menopause (37). Single-cell transcriptomic analysis of OLP lesions here revealed expression of substrate-promoting genes *HMGCR*, *SQLE*, *LSS* and pro-oxidative genes *RELA*, *DUOX1* in multiple clusters, including T cells, with particularly increased levels of *RELA* and *SQLE*, implying a microenvironment conductive to local 7KC accumulation. However, the scores of pro-7-ketocholesterogenic gene set in various tissue cells showed significant downregulation, suggesting suppressed endogenous 7KC generation in tissue-resident cells. Given the demonstrated oxidative stress conditions in OLP lesion and elevated plasma cholesterol levels, which may enhance exogenous 7KC production, the actual 7KC content in OLP tissue requires further exploration (6, 8). Oxysterols can activate LXR receptors and prevent the cleavage activation of SREBP2 into the nucleus (7). 7KC in our study concomitantly stimulated both LXR and SREBP2 expression in OLP T cells, with the latter effect likely attributable to ROS-induced activation of stress pathways that bypass normal sterol regulatory mechanisms (38, 39). Collectively, dysregulated cholesterol metabolites involving in the endocrine-immune disorder exhibited in OLP, with elevated 7KC in OLP plasma, disturbed 7KC biosynthetic capacity in cellular components of OLP tissues, and upregulated cholesterol metabolic regulatory axis SREBP2-LXR induced by 7KC in OLP T cells.

7KC triggered Ca^2+^ influx in T cells, activating calcineurin and subsequently the Ca^2+^-NFATc1 pathway (12). The Ca^2+^-NFATc1 signaling axis was verified aberrantly hyperactive in autoimmune diseases such as rheumatoid arthritis (RA) and SLE, positively correlated with the disease susceptibility and activity in RA (15). Calcineurin inhibitors have shown promising efficacy in autoimmune diseases including OLP by attenuating T cell inflammatory responses and modulating immune cell interactions (15, 27). Our results here demonstrated upregulated Ca^2+^-NFATc1 pathway gene expressions in local OLP T cells, with elevated intracellular Ca^2+^ levels and nuclear translocation of NFATc1 in peripheral OLP T cells. Functional enrichment analysis revealed that OLP T cells with activated calcium signaling were associated with pathways including “Regulation of cell communication by electrical coupling”, “Lipid and atherosclerosis”, “cAMP signaling pathway”, “Amphetamine addiction”, “Oxytocin signaling pathway”, “Aldosterone synthesis and secretion”, “Neurotrophin signaling pathway”, “Dopaminergic synapse” and “Long-term potentiation”. These findings suggest a potential link between Ca^2+^ signaling and lipid metabolism in OLP T cells, and these enriched pathways have been previously demonstrated to regulate T cell migration and tissue homing, Th cell subset polarization, inflammatory oxidative stress, T cell memory formation, and neuroimmune interactions, underscoring the potential roles of Ca^2+^-NFATc1 axis dysregulation in OLP immunopathology (40–48). In addition, 7KC significantly upregulated the levels of Ca^2+^ in primary OLP T cells and OLP plasma-treated Jurkat T cells, as well as the nuclear expression of NFATc1 in primary OLP T cells. Taken together, both peripheral and tissue-infiltrating T cells in OLP exhibited enhanced Ca^2+^-NFATc1 pathway, which was further potentiated by 7KC, and these dysregulated pathways enriched in OLP T cells with hyperactive Ca^2+^-NFATc1 signaling possibly engaged in the OLP pathogenesis by regulating neuro-endocrine-immune interactions linked to T cell migration and pro-inflammatory functions.

The expression of inflammatory cytokines such as MIP-1β, TNF-α, IL-8, IL-1β, IL-6 and the adhesion molecule LFA-1α, coupled with the F-actin cytoskeletal remodeling were confirmed to be promoted by 7KC (21–25). F-actin polymerization positively regulated LFA-1α expression (26). Herein, peripheral OLP T cells exhibited enhanced F-actin polymerization and LFA-1α expression, paralleled by infiltrating T cell transcriptional profiles featuring enriched F-actin dynamics-related gene set and elevated ITGAL. Additionally, OLP T cells with high expression of F-actin dynamics-related gene set and ITGAL showed induced migration signatures such as chemokine genes, adhesion molecule genes, and cytoskeletal regulatory genes, revealing a potential mechanistic link between F-actin and LFA-1α levels with improved migratory and chemotactic capacities of OLP T cells. Our findings further demonstrated that 7KC selectively stimulated migration-related genes *CCL4*/*IL1B*, and inflammatory mediator gene *IL6*, while enhancing LFA-1α for adhesion and F-actin polymerization in primary OLP T cells, without affecting cytotoxic effector genes *TNFA* (encoding TNF-α) or *GZMB* (encoding granzyme B). The contrasting suppression of LFA-1α in Jurkat T cells aligned with the evidenced tendency of this cell line to sacrifice LFA-1α levels for augmented continuous growth which could be stimulated by 7KC (49). Furthermore, 7KC promoted transwell migration of primary OLP T cells and OLP plasma-pretreated Jurkat T cells when co-cultured with LPS-pretreated keratinocytes here, but failed to modulate keratinocyte apoptosis, suggesting selective regulation of migratory over cytotoxic functions by 7KC in OLP. The above results demonstrated that 7KC possibly promoted T cell migration through coordinated upregulations of *CCL4*/*IL1B* genes, F-actin polymerization and LFA-1α expression.

Ca^2+^ influx was validated to drive F-actin polymerization, while blockade of the key immune cell calcium channel CRAC inhibited both LFA-1 activation and transendothelial migration (18, 50). The Ca^2+^-NFAT pathway can also regulate the pro-inflammatory cytokines such as TNF-α, MIP-1β and IL-6 (51–53). This work revealed that blockade of CRAC channels markedly suppressed nuclear expression of NFATc1 in both primary OLP T cells and OLP plasma-treated Jurkat T cells following 7KC treatment. Additionally, it downregulated the expression and polymerization of F-actin, as well as mRNA levels of the core F-actin constituent ACTB and the pro-polymerization molecule-associated gene *DIAPH1*, in both control/OLP primary T cells and control/OLP plasma-treated Jurkat T cells. Moreover, inhibition of CRAC channels reduced the gene expressions of *CCL4*, *IL6* and *IL1B*. As for LFA-1α levels, while calcium channel blockade showed no significant effect on LFA-1α expression in 7KC-primed OLP T cells, the blockade markedly upregulated LFA-1α in Jurkat T cells pretreated by OLP plasma and 7KC. This differential regulation might be explained that 7KC modulated LFA-1α expression in primary T cells through not only Ca^2+^-dependent signaling but also other pathway such as NF-κB pathway, while inhibition of Ca^2+^ pathway potentially relieved the competitive inhibition of LFA-1α expression by activated growth in Jurkat T cells (49, 54). Additionally, Ca^2+^-NFAT pathway blockade significantly reduced the migration of 7KC-pretreated primary OLP T cells when co-cultured with LPS-stimulated keratinocytes. In contrast to the *in vitro* results, scRNA-seq data from OLP lesions revealed downregulated migration-related gene sets in *NFATC1*-high T cells, likely attributable to NFATc1 overexpression-induced exhaustion in tissue infiltrating T cells (55). Overall, our data established that 7KC-dependent modulation of F-actin assembly and expression of *CCL4*, *IL6* and *IL1B* genes were mediated by Ca^2+^-NFATc1 pathway, thereby controlling the migratory capacity of OLP T cells.

**Figure 8:**
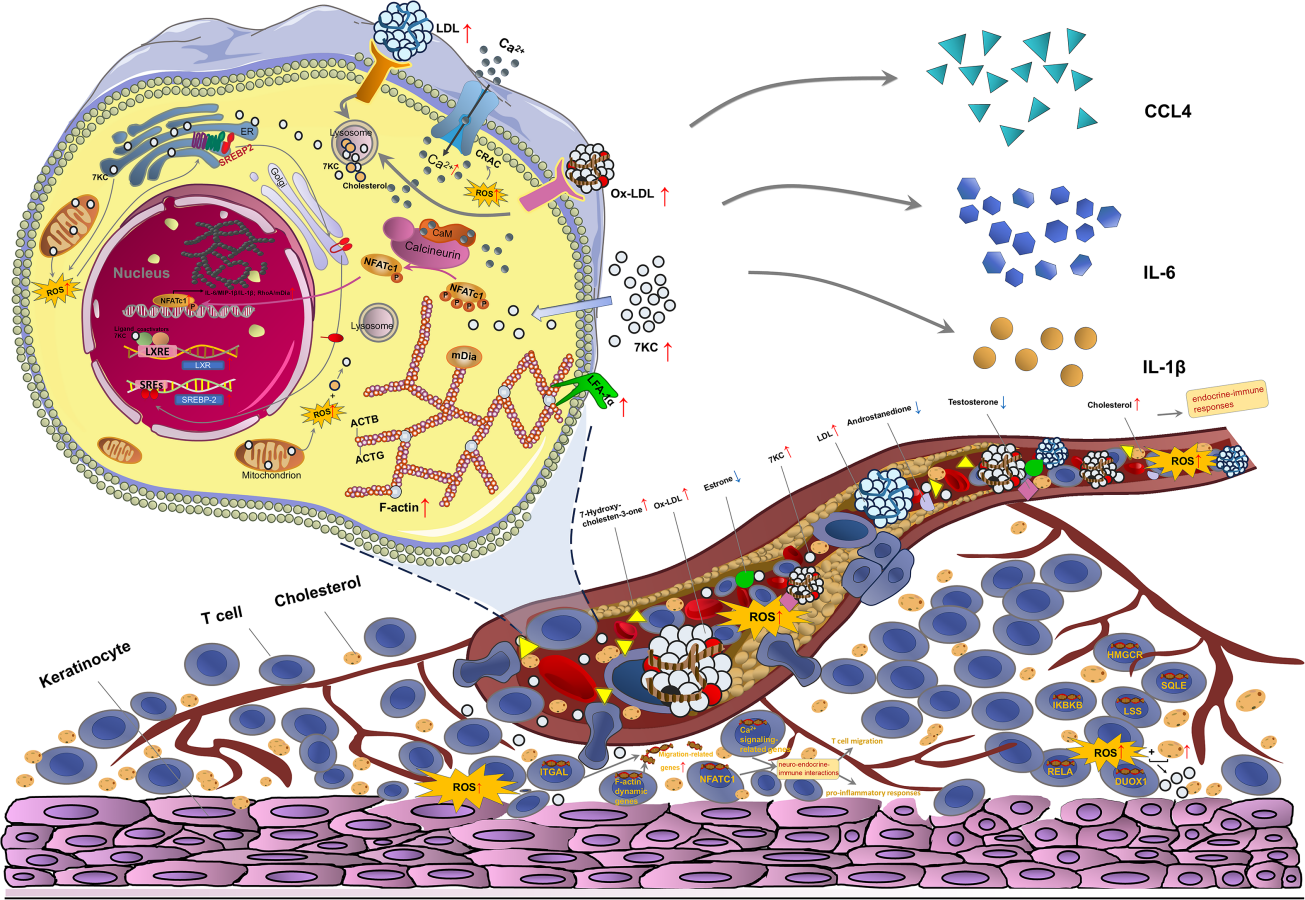
Diagram of 7-ketocholesterol-mediated T cell migration in OLP. Significantly elevated 7-ketocholesterol (7KC) in the disturbed sterol metabolite profile of OLP plasma, combined with dysregulated endogenous 7KC-generating genes and upregulated exogenous ROS-cholesterol pathway in OLP tissue cells, possibly enhance the SREBP2-LXR cholesterol metabolic axis and endocrine-immune responses in OLP T cells. Peripheral and local OLP T cells showed hyperactivated Ca^2+^-NFATc1 signaling, potentiated F-actin polymerization and enhanced LFA-1α, with local OLP T cells exhibiting potentiated F-actin dynamics-related gene sets and expression that positively correlate with migration signatures. Local OLP T cells with hyperactivated Ca^2+^-NFATc1 signaling functionally enrich in neuro-endocrine-immune interactions linked to T cell migration and pro-inflammatory responses. 7KC promotes Ca^2+^ influx, NFATc1 nuclear translocation, and subsequent upregulation of the F-actin component gene *ACTB*, pro-F-actin polymerization molecule-associated gene *DIAPH1* (enconding mDia), adhesion molecule LFA-1α and pro-migratory cytokine genes *CCL4*/*IL1B* along with proinflammatory cytokine gene *IL6*, collectively promoting T cell migration toward keratinocytes. Notably, pharmacological inhibition of Ca^2+^-NFATc1 signaling attenuates 7KC-induced F-actin remodeling via *ACTB*/*DIAPH1* downregulation, reduces inflammatory cytokine secretion, and suppresses T cell migration.

In conclusion, our study provided the first evidence of an aberrant cholesterol-derived steroid profile in OLP plasma, identifying 7KC as a significantly upregulated oxysterol and elucidating its functional role and mechanistic underpinnings in OLP pathogenesis. Gene set associated with 7KC biogenesis was dysregulated in multiple OLP tissue-resident cells. Functional enrichment of abnormal metabolites in OLP plasma highlighted their potential involvement in endocrine-immune dysfunctions. 7KC reciprocally amplified the expression of cholesterol metabolic axis SREBP2-LXR in primary OLP T cells. Notably, the Ca^2+^-NFATc1 pathway was enhanced in both peripheral blood and tissue-infiltrating T cells from OLP patients, with more pronounced activation in NEOLP than EOLP lesions. 7KC potentiated Ca^2+^-NFATc1 signaling in OLP T cells, and the functions of Ca^2+^-NFATc1 pathway-high OLP T cells were enriched in neuro-endocrine-immune interactions linked to T cell migration and pro-inflammatory responses. Additionally, peripheral OLP T cells showed increased F-actin polymerization and LFA-1α expression. Tissue-infiltrating T cells demonstrated upregulation of both F-actin dynamics-related gene set and *ITGAL*, with greater enhancement in NEOLP, positively correlated with migration-associated gene signatures. 7KC enhanced F-actin polymerization, LFA-1α productions, expressions of migration-related cytokine genes *CCL4/IL1B* and proinflammatory cytokine gene *IL6* in OLP T cells, and promoted the migration of OLP T cells in the co-culture system of T cells and keratinocytes. Mechanistically, Ca^2+^-NFATc1 inhibition attenuated 7KC-induced F-actin polymerization, downregulated F-actin regulator genes *ACTB/DIAPH1* and inflammatory cytokine genes *CCL4/IL6/IL1B*, and suppressed OLP T cell migration (**Figure 8**). Given the current lack of standardized animal models for OLP research, our future efforts will focus on developing established murine models and in vivo validation, alongside longitudinal clinical studies and a broader characterization of the role of 7KC within the tissue niche cellular spectrum.

## Data Availability

The raw data supporting the conclusions of this article will be made available by the authors, without undue reservation.
