# Correction: 7-Ketocholesterol promotes T cell migration through Ca2+-NFATc1 pathway-mediated F-actin polymerization and proinflammatory cytokine production in oral lichen planus

**DOI:** 10.3389/fimmu.2026.1817102

**Published:** 2026-03-06

**Authors:** Qin Jiang, Yu-Xi Tang, Gang Zhou

**Affiliations:** 1 State Key Laboratory of Oral & Maxillofacial Reconstruction and Regeneration, Key Laboratory of Oral Biomedicine Ministry of Education, Hubei Key Laboratory of Stomatology, School & Hospital of Stomatology, Wuhan University, Wuhan, China; 2 Department of Oral Medicine, School and Hospital of Stomatology, Wuhan University, Wuhan, China

**Keywords:** 7-Ketocholesterol, Ca^2+^-NFATc1 signaling pathway, F-actin polymerization, oral lichen planus, oxysterol, proinflammatory cytokines

There was a mistake in **Figure 1** as published. A symbol indicating statistical significance in **Figure 1E** was positioned incorrectly. The corrected **Figure 1** appears below.

**Figure 1:**
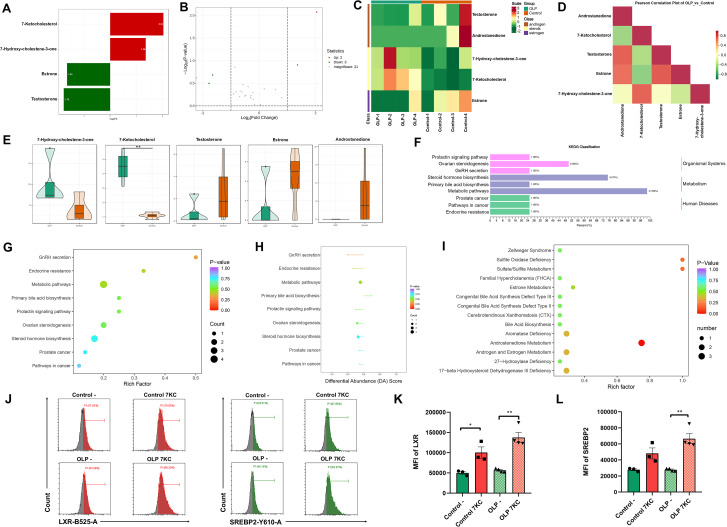
Abnormal steroids in OLP plasma and their functions. **(A–E)**. Steroids in plasma from healthy donors (n=4) and OLP patients (n=4) were determined by LC-MS/MS, and the results of the top 20 metabolites by differential folds were shown as bar chart **(A)**; The results of the top 10 metabolites with the highest FC value were shown in the Volcano diagram **(B)**; Clustering heat map **(C)**; The heat map of correlation between differential metabolites **(D)**; The violin diagram **(E)** of differential metabolites. **(F–I)**. Functional annotation and enrichment analysis of differential metabolites were performed. The bar chart of KEGG classification with the proportion of differential metabolites assigned to each pathway **(F)**, the bubble plots of KEGG functional enrichment **(G)**, differential abundance scores **(H)** and HMDB enrichment analysis **(I)**. **(J–L)**. Flow cytometry was used to determine the levels of cellular cholesterol metabolism regulatory axis SREBP2-LXR in primary T cells treated/untreated with 7-ketocholesterol **(J)**, and the mean fluorescence intensity (MFI) of LXR **(K)** and SREBP2 **(L)** were calculated. Data were presented as mean ± SEM. *p < 0.05, **p < 0.01, 7KC, 7-Ketocholesterol.

The original version of this article has been updated.

