# Supplementary material for: 7-Ketocholesterol promotes T cell migration through Ca2+-NFATc1 pathway-mediated F-actin polymerization and proinflammatory cytokine production in oral lichen planus
Source: Front Immunol. 2026 Feb 6;17:1682589. doi: 10.3389/fimmu.2026.1682589 (PMC12946749; doi:10.3389/fimmu.2026.1682589)
Supplement: Supplementary file 1 [file Table1.docx]

**Supplementary Table 1. The clinical characterizations of the study participants.**

| **Total number** | **OLP patients n = 19** | **Control n = 21** |
| --- | --- | --- |
| **Gender** |  |  |
| Male | 7 | 8 |
| Female | 12 | 13 |
| **Age(years)** |  |  |
| Range | 28-62 | 24-31 |
| Mean±SD | 45.89±10.66 | 27.48±2.06 |
| **Clinical form** |  |  |
| Non-erosive | 8 |  |
| Erosive | 11 |  |
| **RAE scores** |  |  |
| Range | 2-17 |  |
| Mean±SD | 8.24±4.52 |  |
