# Supplementary material for: 7-Ketocholesterol promotes T cell migration through Ca2+-NFATc1 pathway-mediated F-actin polymerization and proinflammatory cytokine production in oral lichen planus
Source: Front Immunol. 2026 Feb 6;17:1682589. doi: 10.3389/fimmu.2026.1682589 (PMC12946749; doi:10.3389/fimmu.2026.1682589)
Supplement: Supplementary file 2 [file Table2.docx]

**Supplementary Table 2. The inclusion and exclusion criteria for OLP patients.**

| **The inclusion criteria:** |
| --- |
| 1. At least 18 years of age and signed written informed consent. |
| 2. Clinically and histopathologically diagnosed with OLP according to the modified WHO diagnostic criteria (van der Meij & van der Waal, 2003) |
| **The exclusion criteria:** |
| 1. A history of smoking or alcohol abuse. |
| 2. Pregnancy, lactation. |
| 3. Subject with infectious, allergic, cardiovascular, hematological, endocrine, metabolic, and immune-related diseases. |
| 4. Exposure to systemic or topical anti-inflammatory, immunomodulatory drugs at least within 3 months. |
| 5. Patient with concomitant other oral lesions. |
| 6. Participant with oral lichenoid reactions, including lichenoid contact reactions, lichenoid drug eruptions, and lichenoid reactions of graft- versus host disease. |
| 7. Presence of epithelial dysplasia in histopathological examination |
