# Supplementary material for: 7-Ketocholesterol promotes T cell migration through Ca2+-NFATc1 pathway-mediated F-actin polymerization and proinflammatory cytokine production in oral lichen planus
Source: Front Immunol. 2026 Feb 6;17:1682589. doi: 10.3389/fimmu.2026.1682589 (PMC12946749; doi:10.3389/fimmu.2026.1682589)
Supplement: Supplementary file 3 [file Table3.docx]

**Supplementary Table 3. RAE scoring system for OLP.**

| **Clinical signs** | **Scoring** |
| --- | --- |
| Reticular lesions (R) | 0 = no white striations |
|  | 1 = presence of white striations or keratotic papules |
| Atrophic areas (A) | 0 = no lesion |
|  | 1 = lesions less than 1 cm^2^ |
|  | 2 = lesions from 1 to 3 cm^2^ |
|  | 3 = lesions greater than 3 cm^2^ |
| Erosive areas (E) | 0 = no lesion |
|  | 1 = lesions less than 1 cm^2^ |
|  | 2 = lesions from 1 to 3 cm^2^ |
|  | 3 = lesions greater than 3 cm^2^ |
| The total score of all 10 areas | ΣR + Σ(A × 1.5) + Σ (E × 2.0) |

Note: The oral cavity of each individual was divided into 10 sites: upper/lower labial mucosa, right buccal mucosa, left buccal mucosa, dorsal tongue, ventral tongue, floor of mouth, hard palate mucosa, soft palate/tonsillar pillars, maxillary gingiva, mandibular gingiva.

Abbreviations: OLP, oral lichen planus; RAE, reticular, atrophic, erosive.
