# Supplementary material for: 7-Ketocholesterol promotes T cell migration through Ca2+-NFATc1 pathway-mediated F-actin polymerization and proinflammatory cytokine production in oral lichen planus
Source: Front Immunol. 2026 Feb 6;17:1682589. doi: 10.3389/fimmu.2026.1682589 (PMC12946749; doi:10.3389/fimmu.2026.1682589)
Supplement: Supplementary file 4 [file Table4.docx]

**Supplementary Table 4. List of defining gene sets in the analysis of RNAseq data.**

| **Pro-7-ketocholesterogenic gene set** | **Calcium signaling pathway gene set** | **F-actin dynamics gene set** | **Migration gene set** |
| --- | --- | --- | --- |
| CYP7A1 | ORAI1 | ACTB | CXCR3 |
| HMGCR | STIM1 | ACTG1 | CCR5 |
| SQLE | STIM2 | ARPC1 | CCR4 |
| LSS | TRPC1 | ARPC2 | CXCR4 |
| NOX1 | IP3R | ARPC3 | ITGAL |
| NOX2 | CALM1 | ARPC4 | ITGB2 |
| NOX3 | CALM2 | ARPC5 | ITGA4 |
| NOX4 | CALM3 | WAS | ITGB1 |
| NOX5 | PPP3CA | WASF1 | ITGB7 |
| DUOX1 | CAMK2D | WASF2 | STAT1 |
| DUOX2 | CAMK2G | WASF3 | STAT4 |
| NFKB1 | CAMK4 | FMN1 | PIK3CD |
| RELA | NFATC1 | FMN2 | AKT1 |
| IKBKB | NFATC2 | PFN1 | RHOA |
| HIF1A | NFATC3 | PFN2 | CDC42 |
| ALOX15 | NFATC4 | PLS3 | CD44 |
| ALOX12 | NFAT5 | ACTN1 |  |
|  | CREB1 | ACTN4 |  |
|  | IL2 | DIAPH1 |  |
|  | CD69 | RHOA |  |
|  | FOS | RAC1 |  |
|  | JUN | CDC42 |  |
|  |  | MYH9 |  |
