# Supplementary material for: 7-Ketocholesterol promotes T cell migration through Ca2+-NFATc1 pathway-mediated F-actin polymerization and proinflammatory cytokine production in oral lichen planus
Source: Front Immunol. 2026 Feb 6;17:1682589. doi: 10.3389/fimmu.2026.1682589 (PMC12946749; doi:10.3389/fimmu.2026.1682589)
Supplement: Supplementary file 5 [file Table5.docx]

**Supplementary Table 5. Primers designed for quantitative real-time PCR**

|  | Forward | Reverse |
| --- | --- | --- |
| IL8 | 5′-CTCTTGGCAGCCTTCCTGATTTC-3′ | 5′-GGGTGGAAAGGTTTGGAGTATGTC-3′ |
| IL6 | 5′-AGAGTAGTGAGGAACAAGCCAGAG-3′ | 5′-GGCATTTGTGGTTGGGTCAGG-3′ |
| IL1B | 5′-GCACCTGTACGATCACTGAACTG-3′ | 5′-CACTTGTTGCTCCATATCCTGTCC -3′ |
| TNFA | 5′-CTCATCTACTCCCAGGTCCTCTTC-3′ | 5′-CGATGCGGCTGATGGTGTG-3′ |
| ITGAL | 5′-AGCCAAGTCAGCGGATAGAAGG-3′ | 5′-GCCACATCTGCCAAGCCATC-3′ |
| CCL4 | 5′-ATGAAGCTCTGCGTGACTGTCC-3′ | 5′-AGGCGGTGGGAGGGTCTG-3′ |
| GZMB | 5′-TGCGGTGGCTTCCTGATACG-3′ | 5′-TCGGCTCCTGTTCTTTGATATTGTG-3′ |
| ACTB | 5′-ATTGCCGACAGGATGCAGAA-3′ | 5′-CGGACTCGTCATACTCCTGC-3′ |
| ACTG | 5′-TGTTTCCTTCCATCGTCGGG-3′ | 5′-CATGTCGTCCCAGTTGGTGA-3′ |
| DIAPH1 | 5′-GCCAAACTGGTCCAAGCTTG-3′ | 5′-TGTTCTCAAAGCGGTCCTCC-3′ |
| NFATC1 | 5′-CCAGTACCAGCGTTTCACCT-3′ | 5′-GACTTAACCCCTGGCTCACC -3′ |
| GAPDH | 5′-CGGAGTCAACGGATTTGGTCGTAT-3′ | 5′-AGCCTTCTCCATGGTGGTGGTGAAGAC-3′ |
