# Supplementary material for: 7-Ketocholesterol promotes T cell migration through Ca2+-NFATc1 pathway-mediated F-actin polymerization and proinflammatory cytokine production in oral lichen planus
Source: Front Immunol. 2026 Feb 6;17:1682589. doi: 10.3389/fimmu.2026.1682589 (PMC12946749; doi:10.3389/fimmu.2026.1682589)
Supplement: Supplementary file 6 [file Table6.docx]

**Supplementary Table 6.** **HMDB-annotated differential metabolites-associated diseases.**

| Compound Name | HMDB Diseases |
| --- | --- |
| 7-Hydroxy-cholesten-3-one | Alcoholism / Crohn’s disease / Gallbladder disease / Irritable bowel syndrome / Diverticular disease / Ileocystoplasty / Meckels diverticulum / Atrophic gastritis / Chronic pancreatitis |
| 7-Ketocholesterol | Multiple sclerosis |
| Testosterone | 11-beta-Hydroxylase deficiency / 21-Hydroxylase deficiency / Adrenal hyperplasia, congenital, due to 3-beta-hydroxysteroid dehydrogenase 2 deficiency / Cadmium exposure / Schizophrenia / Hypogonadism / Prostate cancer / Congenital Adrenal Hyperplasia, due to 17-Hydroxylase-Deficiency / Adrenal insufficiency, congenital, with 46,XY sex reversal, partial or complete / Aromatase deficiency / Lipoid Congenital Adrenal Hyperplasia / Woodhouse-Sakati syndrome / X-linked ichthyosis |
| Estrone | Menstrual cycle / Aromatase deficiency / X-linked ichthyosis |
| Androstanedione | - |
